# Reduced expression of α-L-Fucosidase-1 (FUCA-1) predicts recurrence and shorter cancer specific survival in luminal B LN+ breast cancer patients

**DOI:** 10.18632/oncotarget.24445

**Published:** 2018-02-07

**Authors:** Serena Bonin, Alessia Parascandolo, Cinzia Aversa, Renzo Barbazza, Nobuo Tsuchida, Maria Domenica Castellone, Giorgio Stanta, Giancarlo Vecchio

**Affiliations:** ^1^ Dipartimento di Scienze Mediche, Università di Trieste-Cattinara, Trieste, Italy; ^2^ IRCSS SDN, Naples, Italy; ^3^ Graduate School of Medical and Dental Sciences, Tokyo Medical and Dental University, Tokyo, Japan; ^4^ Istituto di Endocrinologia e Oncologia Sperimentale G.Salvatore, CNR, Naples, Italy; ^5^ Dipartimento di Medicina Molecolare e Biotecnologie Mediche, Università di Napoli Federico II, Naples, Italy; ^6^ Istituto Superiore di Oncologia, Naples, Italy; ^7^ Istituto Superiore di Oncologia, Genoa, Italy

**Keywords:** breast cancer, luminal B, lymph node metastasis, α-L-Fucosidase-1, immunohistochemistry

## Abstract

**Background:**

The lysosomal enzyme α-L-Fucosidase-1 (FUCA-1) catalyzes the hydrolytic cleavage of terminal fucose residues. FUCA-1 gene is down-regulated in highly aggressive and metastatic human tumors as its inactivation perturbs the fucosylation of proteins involved in cell adhesion, migration and metastases.

**Results:**

Negativity to FUCA-1 was significantly related to the development of later recurrences in breast cancer patients with lymph node involvement at diagnosis. Cancer specific survival of luminal B LN+ patients was influenced by FUCA-1 expression as luminal B LN+ patients with positive expression had a longer cancer specific survival. FUCA-1 mRNA expression was inversely related to cancer stage and lymph node involvement. WB and qPCR analysis of FUCA-1 expression in breast cancer-derived cell lines confirmed an inverse relationship with tumor aggressiveness.

**Conclusions:**

This study shows that, within LN+ breast cancer patients, FUCA-1 is able to identify a sub-set of non recurrent patients characterized by the positive expression of FUCA-1 and that, within luminal B LN+ patients, the expression of FUCA-1 predicts longer cancer specific survival.

**Methods:**

We have analyzed FUCA-1 in 305 breast cancer patients by Immunohistochemistry (IHC), and by qPCR in breast cancer patients and in breast cancer cell lines.

## INTRODUCTION

Breast cancer (BC) is the most frequent female neoplasia representing the first cause of women cancer death (data from http://globocan.iarc.fr/) and a heterogeneous group of tumors both at the molecular and morphological levels. Also the clinical course of the disease is highly variable, at the same tumor grade and stage; some patients are completely cured, while others recur, even 10 years after surgery. In the last decade, mammary carcinomas have been classified molecularly in 5 main subtypes: three estrogen receptor positive (luminal A and B and HER2+ luminal), one HER2+ non luminal and the triple negative (TN). These subtypes have different prognosis and clinical course [1, 2]. Luminal tumors (both A and B) include more than two thirds of all breast cancers [3]. Although they have a better prognosis, patients with luminal tumors may recur and die from the disease even more than 10 years after surgery [4]. Besides the molecular classification, the presence of lymph-nodal metastasis at diagnosis is one of the most relevant prognostic factors for diagnosis of BC patients. Lymph-node negative patients have 75% survival probability at 20 years of follow-up, while lymph-node positive patients have 40% survival, similar to TN patients [4]. Therefore, the possibility to detect new biomarkers for a more specific prognosis in the latter group of patients would be relevant.

Previous observations, regarding the role of fucosylation in cancer, indicated that the human lysosomal enzyme α-L-fucosidase-1 (FUCA-1, EC number 3.2.1.51) is down-regulated in highly aggressive human tumors such as neuroblastomas [5], breast [6], and colorectal cancers [7]. Since elevated fucose levels are preferentially expressed in metastatic foci versus primary tumors [8–10], it has been suggested that the study of altered fucose in tumor cells could be useful for searching new treatment targets [11]. Yuan *et al.* [11] hypothesized that a decrease of fucose content might alter the biological behavior of breast cancer cells, and, especially, the interaction among tumor cells, the ECM and endothelial cells, yielding new information for diagnosis and treatment of metastases.

We recently reported [12] that FUCA-1 is expressed at normal levels in less aggressive, differentiated papillary thyroid cancers, whereas it is down-regulated in highly malignant, anaplastic thyroid cancers compared with its expression in normal thyroid tissues. In this work we studied FUCA-1 expression in breast cancer aggressiveness and prognosis with respect to lymph node involvement.

We report here that negativity to FUCA-1 is significantly related to the development of later recurrences in breast cancer patients with lymph node involvement at diagnosis. Furthermore, higher expression of FUCA-1 among luminal B LN+ patients can identify a sub-group of patients with a better outcome.

## RESULTS

### Antibody specificity

**Figure 1:**
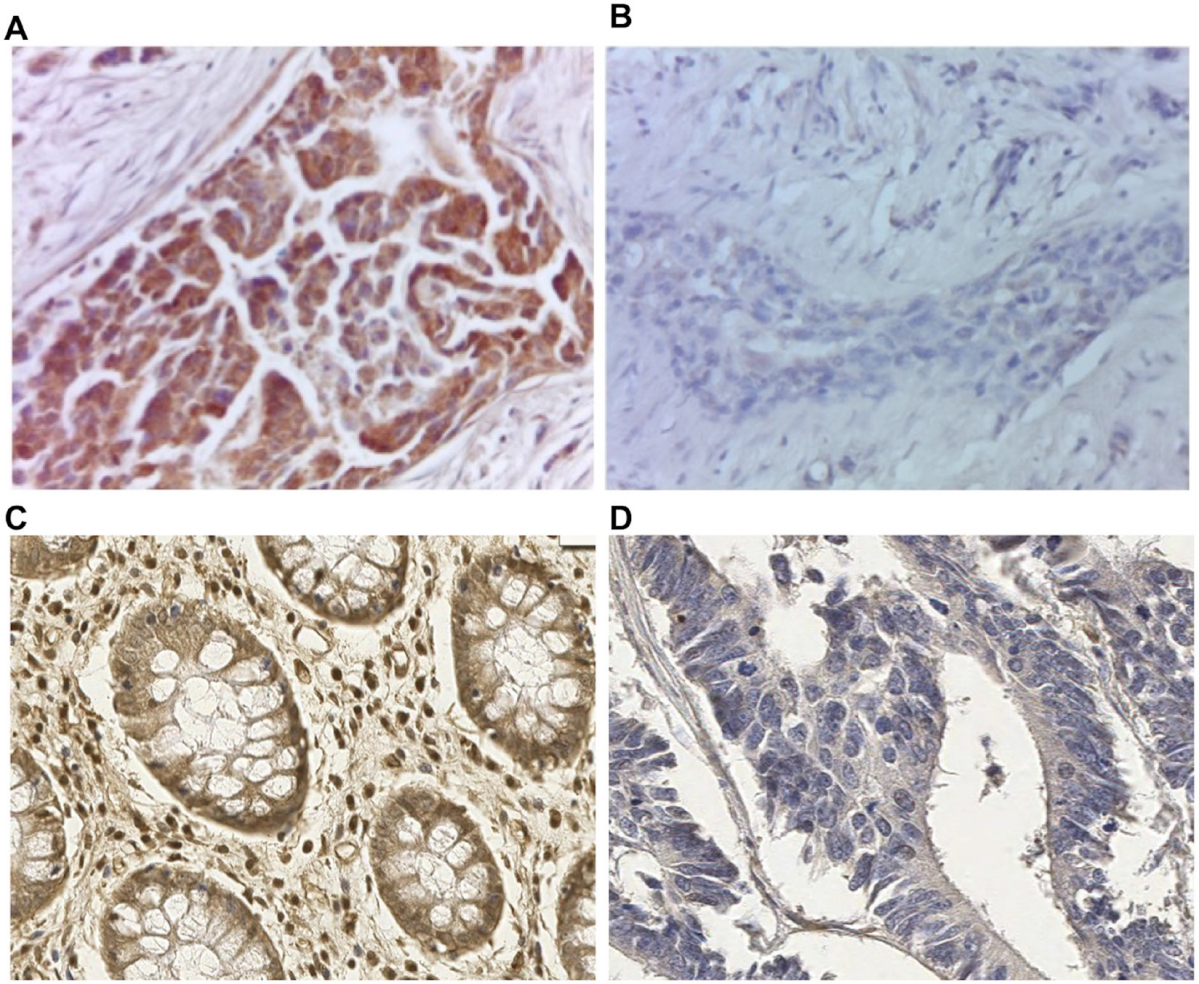
Preabsorption test to verify FUCA-1 polyclonal antibody specificity on luminal A breast cancer lesion Immunostaining with the FUCA-1 antibody in breast cancer tissue (**A**) and after preabsorption with a cell extract obtained from a continuous thyroid papillary carcinoma cell line (TPC-1) expressing high levels of the FUCA-1 protein (**B**). Magnification: 40×; conventional immunohistochemistry performed with di-aminobenzidine as chromogen and hematoxylin as counterstaining. Positive immunostaining on human normal colon mucosa (**C**) and negative control on colon adenocarcinoma (**D**) with the same FUCA-1 antibody, Magnification 20×.

The specificity of the FUCA-1 signal detected by IHC was assessed by the pre-absorption test (Figure 1A, 1B). The positive staining pattern was clearly evident in a histological section of luminal breast cancer (Figure 1A). However, as reported in Figure 1B, the staining pattern was completely eliminated after incubation of the antibody with the protein extract of TPC-1 cells expressing high levels of the FUCA-1 protein. As reported in Figure 1C and 1D, the immunohistochemical staining was positive in normal colon mucosa, and negative in colon adenocarcinoma. Results were considered negative if no staining was detectable or if staining was present in less than 10% of the cells examined.

### Expression levels of FUCA-1 in different histotypes of breast cancers

Immunohistochemical stainings with the FUCA-1 antibody in normal mammary gland and breast carcinomas with different levels of differentiation are shown in Supplementary Figure 1.

Well-differentiated tumor samples showed a higher number of positive cells for FUCA-1 compared to poorly differentiated tumors.

### Patients

Mean age of patients at diagnosis was 47 years (range 26–55). During the period of observation 18 patients were lost at follow-up because of emigration during follow-up. At diagnosis 154 patients presented axillary lymph node involvement while 151 did not. No significant differences in age at diagnosis were observed between the LN− (46.8 y, range 32–55 y) and LN+ (47.0 y, range 26–55 y) groups, but the frequency of patients under 35 years was higher in LN+.

The median follow-up time was 16 years (range 0–25). In detail, it was 18 years (range 0–25) for the LN− group and 9 (range 0–24) for the LN+ group (*p* = 0.000). In the LN− group 45 women (31%) recurred, while in the LN+ group 98 patients (67%) did so. For 4 LN− patients (3%) and 11 LN+ ones (7%) no information about recurrences was available. All clinical and pathological characteristics are reported in Supplementary Table 1.

### Relationship between FUCA-1 expression and clinico-pathological features

**Figure 2:**
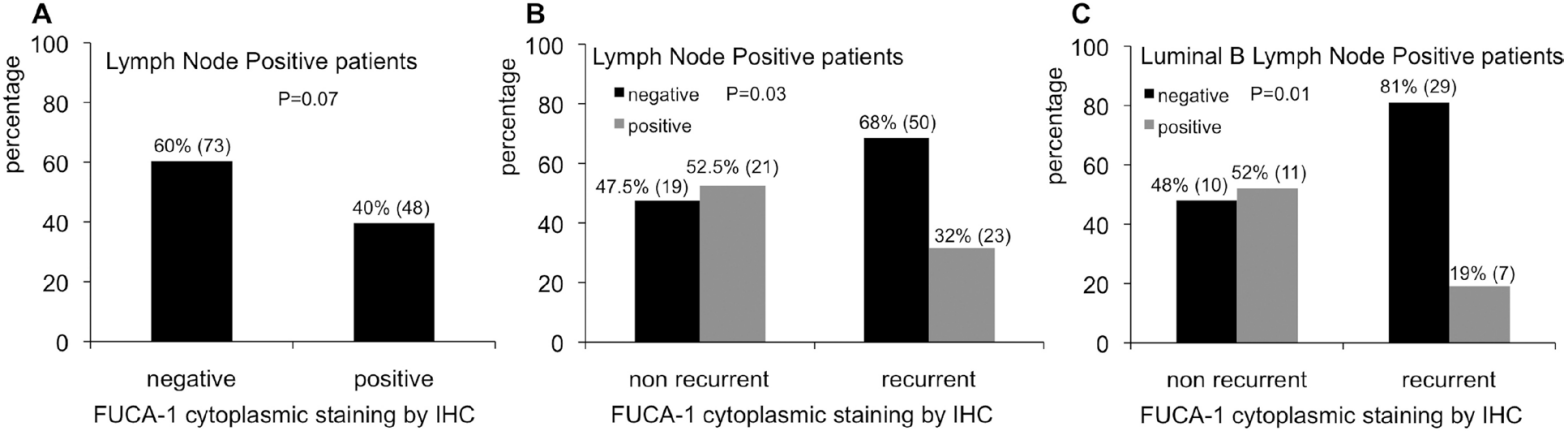
Relative percentages of FUCA-1 immunostaining in LN+ patients (**A**). Relative percentages of FUCA-1 immunostaining in non recurrent and recurrent LN+ patients (**B**). Relative percentages of FUCA-1 immunostaining in non recurrent or recurrent luminal B LN+ patients (**C**).

The relationship between FUCA-1 expression and clinico-pathological features was evaluated in 204 patients since cores were not always analyzed for technical reasons in the remnants. The results are reported in Figure 2A. The expression of FUCA-1 in BC patients with lymph node positive cancers, considering all molecular subtypes, was negative in 60% of patients against 40% being positive, thus suggesting that the lack of expression of FUCA-1 correlates with a more aggressive clinical behaviour of the tumor.

### Relationship between FUCA-1 expression, recurrences frequencies and molecular subtypes

BC primary tumors were classified as follows: 83 luminal A, 77 luminal B, 20 HER-2+, 24 TN subtypes and 1 unclassified. The expression of FUCA-1 was not associated to individual subtypes (*p* = 0.9), except for lymph node positive patients. Considering all molecular subtypes of LN+ patients taken together, the lower expression of FUCA-1 was associated to the development of later recurrence (Figure 2B). Negativity to FUCA-1 expression, in fact, was significantly related to the development of later recurrences (*p* = 0.03), since 68% of recurrent patients were negative to FUCA-1, compared to 47% of non recurrent ones (Figure 2B). This trend was even more evident when only the group of luminal B lymph node positive patients was analysed, 81% of whom were negative for FUCA-1 expression, as shown in Figure 2C (*p* = 0.01). This result was not confirmed in lymph node negative patients (*p* = 0.5) (data not shown).

### FUCA-1 mRNA expression

**Figure 3:**
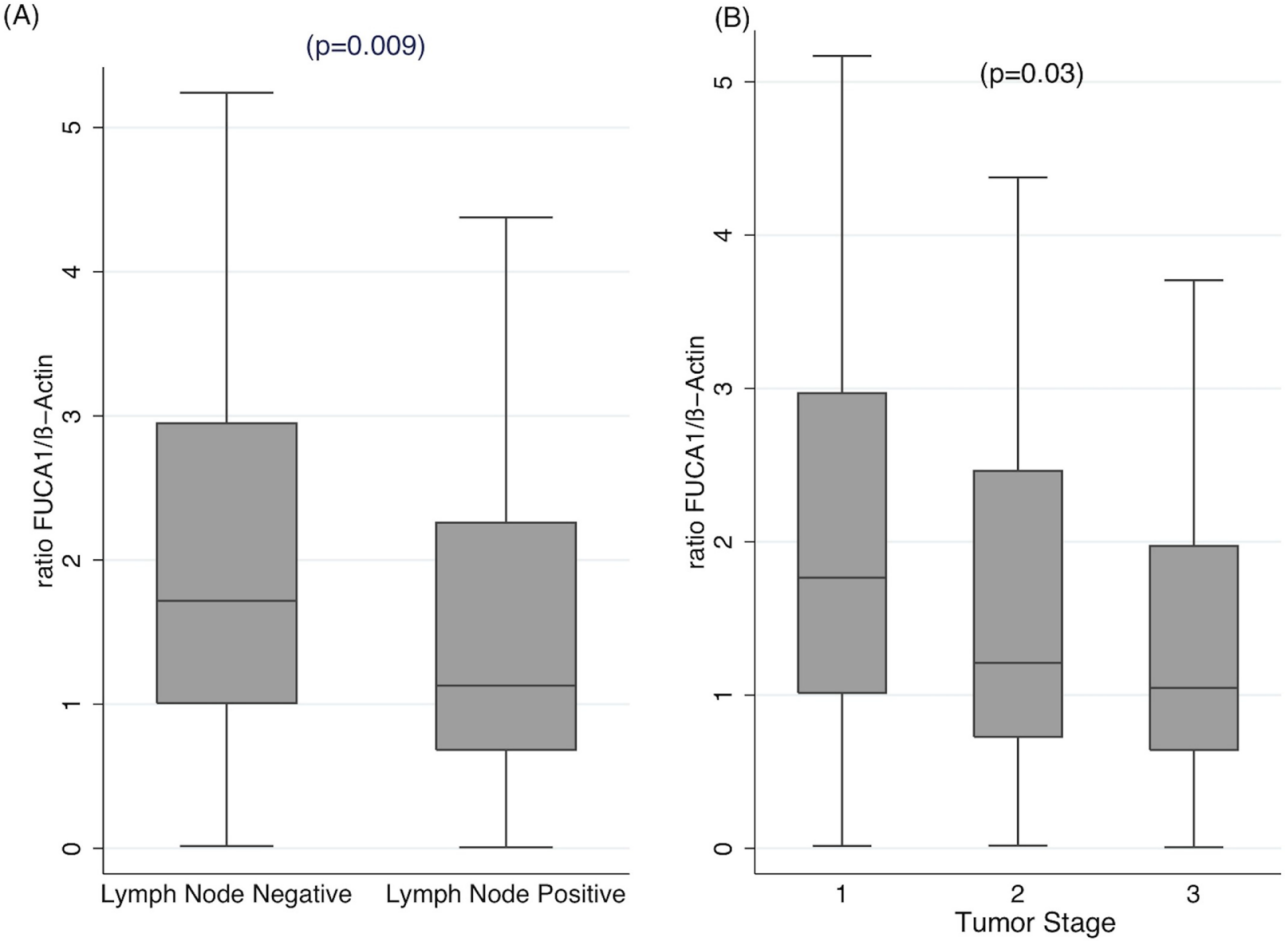
qRT-PCR of FUCA-1-specific RNA extracted from FFPE tissues of the same cohort of patients analyzed in Figures 2, 4 and 5 The relative ratios of FUCA-1 mRNA to β-actin mRNA is plotted against LN+ or LN− patients (**A**) or in patients with different clinical stage (**B**).

To confirm the relationship between aggressiveness of breast cancer and expression of FUCA-1, we have analyzed by RT-qPCR FUCA-1-specific mRNA expression in the cohort of LN+ and LN− patients. Figure 3A shows that LN+ patients display significantly lower levels of FUCA-1 specific mRNA. Similarly, Figure 3B shows that FUCA-1 mRNA expression is inversely related to BC tumor stage.

### Relationship between FUCA-1 expression and survival

At the end of follow-up 87 women were alive, 14 died of any cause different from BC, and 90 patients died from breast carcinoma. Thirteen patients were lost at follow-up.

**Figure 4:**
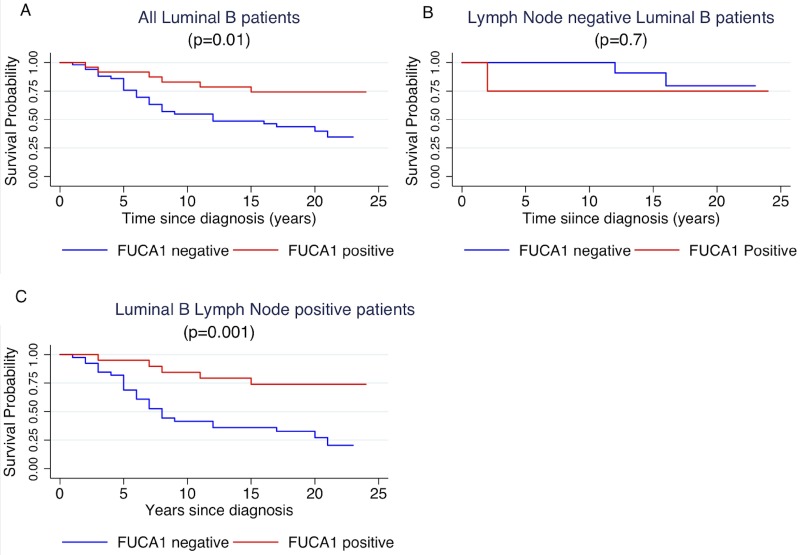
Kaplan- Meier curves of cancer specific survival by positivity or negativity to FUCA-1 immunostaining in all luminal B patients (**A**), in LN– luminal B patients (**B**) and in LN+ luminal B patients (**C**).

Cancer specific survival of patients was investigated per molecular subtypes with respect to FUCA-1 positivity, as reported in Table 1. Survival of Luminal B patients was influenced by FUCA-1 cytoplasmic staining, as shown in Figure 4A (*p* = 0.01), where a longer cancer specific survival in patients expressing FUCA-1 is evident. After separating the patients’ cohort, according to lymph node involvement, luminal B LN+ patients with a FUCA-1 positive expression had a significantly longer specific survival (*p* = 0.001) (Figure 4C), whereas luminal B LN− women did not (*p* = 0.7; Figure 4B).

**Table 1:** Results of FUCA-1 IHC analysis (*p* values refer to Chi2 test and to *t*-test for age at diagnosis)

			FUCA-1 IHC Negative	FUCA-1 IHC Positive	*p*
**Clinical-pathological features**
	**Age, mean, years**		47	47.4	0.6
	**Tumor Stage**	1	39	20	0.9
		2	60	30	
		3	34	20	
	**Tumor grade**	G1	13	11	0.4
		G2	59	33	
		G3	61	27	
	**Lymph Node Involvement**	No	60	23	**0.07**
		Yes	73	48	
	**Histotype**	Ductal	106	61	0.1
		Lobular	15	3	
		Medullary	5	0	t
		Mucinous	3	4	
		Tubular	4	3	
	**Later recurrences**	No	52	36	0.1
		Yes	74	31	
	**Molecular subtypes**	Luminal A	54	29	0.9
		Luminal B	51	24	
		HER2	12	8	
		TN	15	9	
	**Number of Positive LN**	≤3	46	31	0.7
		>3	27	13	

**Figure 5:**
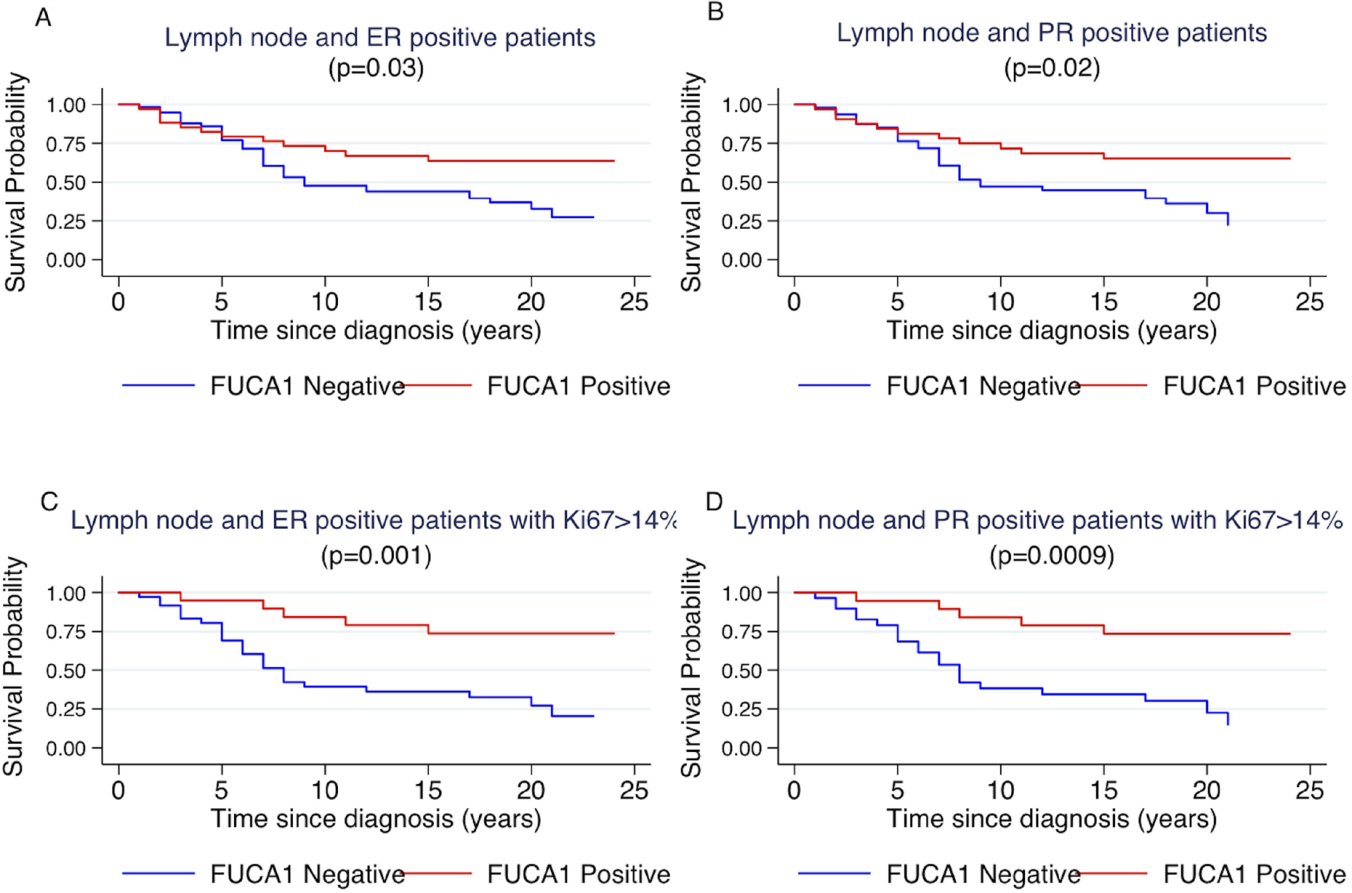
Kaplan-Meier curves of cancer specific survival by positivity or negativity to FUCA-1 immunostaining in LN+ ER+ patients (**A**), in LN+, PR+ patients (**B**), in LN+, ER+ patients with Ki67 staining higher than 14% (**C**) and in LN+, PR+ patients with Ki67 staining higher than 14% (**D**).

However, considering the entire cohort of patients, without molecular sub-type grouping, there was not significant difference between FUCA-1 expression in LN− (*p* = 0.1) and LN+ patients (*p* = 0.3). To better explore the relationship between luminal B patients and FUCA-1 expression, we have investigated the survival curves of lymph node positive patients with respect to the positivity of the surrogate markers that define the luminal B subtype, i.e., positivity to oestrogen and/or progesterone receptor and Ki67 positivity in more than 14% of cells. Patients survival was influenced by FUCA-1 expression in LN+ expressing ER+, PR+ (Figure 5A and 5B). The effect of FUCA-1 was even more evident by examining patients with ER+, PR+ and a Ki67 value higher than 14 % (see Figure 5C and 5D). The same analysis carried out in the LN− subgroup did not show significant differences as reported in the Supplementary Figure 2. Longer cancer specific survival was also observed for Luminal B- HER 2+ patients expressing FUCA-1 in comparison with those who did not express it (Supplementary Figure 3).

Cox regression analysis for luminal B LN+ patients using as covariates stage, grade, age at diagnosis, histological type of tumors and FUCA-1 cytoplasmic staining by IHC (Regression *p* = 0.04) confirmed the protective effect of FUCA-1 (HR= 0.25, 95% CI 0.09–0.69 *p* = 0.008) as reported in Supplementary Table 2. Results on the effect of cytoplasmic FUCA-1, Ki67, ER, PR on survival in our cohort of LN+ BC patients are summarized in Supplementary Table 3, where *p* values refer to log-rank test obtained for cytoplasmic expression of FUCA-1 in LN+ BC patients positive for the marked biomarkers.

### FUCA-1 protein and mRNA expression in breast cancer cell lines of different molecular subtype

**Figure 6:**
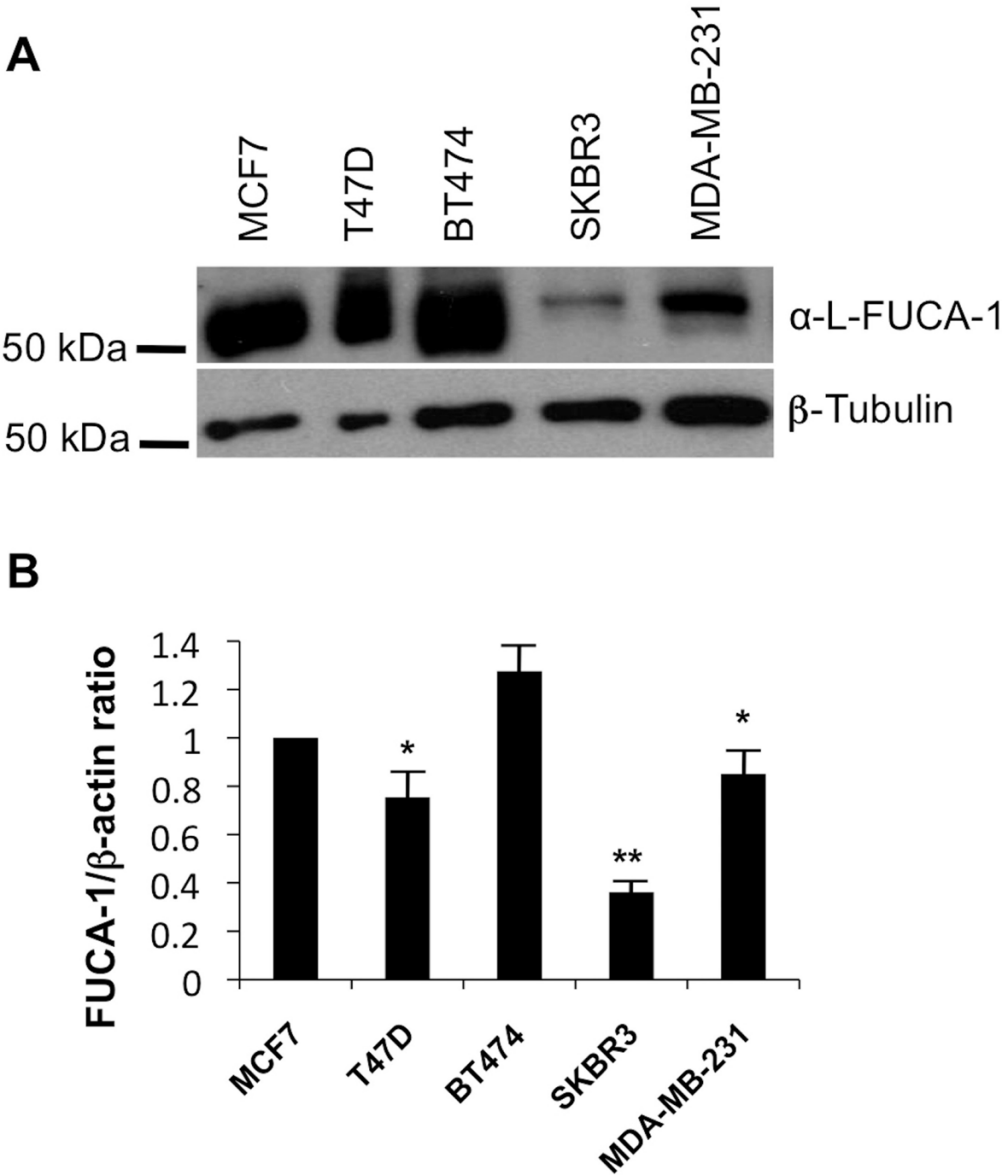
WB and qPCR in breast cancer cell lines extracts Western blot analysis was performed on a panel of breast cancer cells including MCF7 (luminal A), T47D (Luminal A), BT474 (Luminal B), SKBR3 (HER+), MDA-MB231 (TN, claudin low) using an anti FUCA-1 antibody and normalized by β tubulin expression (**A**). Relative expression of FUCA-1 (FUCA-1/β-actin ratio) calculated upon comparison with FUCA-1 expression in MCF7 (luminal A) cells, normalized on beta actin expression is also shown (**B**).

FUCA-1 protein and mRNA expression was analyzed in a panel of breast cancer cell lines established from patients with different histotypes of BC tumors (Figure 6). In particular, we analyzed the MCF7 (luminal A), the T47D (luminal A), the BT474 (luminal B), the SKBR3 (HER2+, ER-, PR-) and the MDA-MB231 (claudin low, triple negative) [13] cell lines by WB and qPCR finding that expression of FUCA-1 was strongly decreased in less differentiated molecular subtypes SKBR3 and in the triple negative subtype MDA-MB231. Furthermore, the analysis of a publically available database (Oncomine) (see Supplementary Table 4) shows that a lower expression of FUCA-1 mRNA is characteristic of histologically more invasive and aggressive breast cancer cell lines subtypes.

## DISCUSSION

α-L-fucosidases are exoglycosidases widespread in nature [14], ubiquitously expressed in eukaryotic cells. In humans the FUCA-1 gene, coding for the lysosomal enzyme α-L-fucosidase-1, has been widely studied [15]. In this study, we investigated the expression of FUCA-1 both at mRNA and protein levels in breast cancer (BC). Our results thus showed clearly that negativity to FUCA-1 is significantly related to the development of later recurrences in breast cancer patients with lymph node involvement at diagnosis. Moreover, cancer specific survival of luminal B LN+ patients was influenced by FUCA-1 expression, since these patients, having positive FUCA-1 expression, had a longer survival. Our results thus showed clearly that FUCA-1 is able to define a sub-group of luminal B, lymph node positive patients with a favorable prognosis and that this protein may represent a positive marker to discriminate a subset of LN+ patients who will have a lower risk of recurrence and longer cancer specific survival.

Other authors have studied the role of FUCA-1 in BC. Milde-Langosh *et al.* [16] identified in BC FUCA-1 among 24 relevant genes, coding for sixteen anabolic and eight catabolic enzymes, with independent prognostic value. Low FUCA-1 expression correlated significantly with a shorter relapse free survival as well as overall survival, while FUCA-1 overexpression was associated with a relatively good outcome, showing an independent prognostic value, in agreement with the present results. Furthermore, FUCA-1 was shown to be up-regulated only in Luminal A breast cancer patients compared to basal-like breast cancer patients in a study of glycan-related gene expression profiling in breast cancer subtypes [17]. These authors however did not examine differences of FUCA-1 expression between LN+ and LN− patients. A decreased expression of the FUCA-1- gene was also found in human colorectal carcinomas, compared to normal mucosa and a gradual decrement in FUCA-1 expression was observed with progression of the disease from earlier to advanced stages [7]. Furthermore, a reduction in the α-L-Fucosidase activity in the tumor vs. normal mucosa has been correlated with recurrence in more than 52% of colorectal carcinoma patients and was considered as a good independent prognostic factor [18].

The role of FUCA-1 in reducing invasiveness in breast and colon cancer could be explained by FUCA-1 mediated decrease in the composition and quantity of cell surface fucosylation-associated molecules [19]. In line with Cheng *et al.* (2015) [19], who detected FUCA-1 in early stage BC, our findings showed an inverse association between FUCA-1 mRNA and tumor stage. The expression of the FUCA-1 gene has been reported to be directly controlled by p53 [20, 21]. Mutated p53 has been associated with lower expression of FUCA-1 in a set of human thyroid cancer cell lines [21]. In this respect, it is interesting to notice that mutations in p53 represent the most common genetic alteration in breast cancer, found in average 30% of breast tumors, with higher frequencies in most aggressive cancers (50% of HER2 amplified BC and 88% of basal-like carcinomas) whereas its frequency is lower in luminal tumors (17% of luminal A, 41% of luminal B) [22].

Thus, it is likely that within luminal B tumors with LN + higher FUCA-1 expression is associated with the wild type p53, thereby giving good prognosis.

Furthermore, some markers, such as E-cadherin, CD44 and CD24, which characterize the metastatic potential of human breast cancer cells, have been related to the molecular subtypes. It has been demonstrated that low colony forming activity of human breast cancer cells of luminal subtype is related to increased adhesive properties of these cells, whereas high tumorigenicity of cells of basal subtype is connected to weakening of adhesive contacts [23]. These observations are in line with our results on BC cell lines showing an inverse relationship between FUCA-1 expression and aggressiveness, since claudin low triple-negative (MDA-MB231) and HER2 overexpressing (SKBR3) BC cell lines showed lower expression of FUCA-1 in comparison to luminal A ones (MCF-7 and T47D) and luminal B (BT474) both by WB and qPCR.

Our results are also in agreement with those of Ezawa *et al.* [24], who found that lower FUCA-1 expression was associated with poor prognosis in cancer patients, especially in colorectal and breast cancer patients.

FUCA-1 is a marker of good prognosis in several types of tumors. In the absence of routine gene expression profiling, surrogate IHC markers for molecular breast cancer subtypes represent a more practical means of characterizing BC tumor types according to prognosis and/or differential response to specific agents [25, 26]. Moreover, the success of new anti-cancer therapies is likely to be dependent upon the use of new biomarkers to detect patients who will benefit from a particular treatment [26]. In this study we have shown that FUCA-1 is able to define a subset of luminal B lymph node positive BC with longer cancer specific and overall survival. Our findings are relevant because most existing biomarkers are predictive or prognostic in lymph node negative BC. Our data clearly show that FUCA-1 may represent a reliable biomarker for clinical use easily detectable by immunohistochemistry staining. Larger multicenter studies will be necessary to confirm the use of FUCA-1 in clinical practice.

## MATERIALS AND METHODS

### Patients

All patients were resident in a Northern-East province of Italy and were already examined in a previous study [4]. Inclusion criteria were: i) diagnosis of BC at least 15 years before the censoring date of the study (31st December 2008), ii) invasive BC of stage I-III, iii) age at diagnosis 55 years or younger, iv) availability of formalin-fixed and paraffin-embedded (FFPE) tissues. Cases with a second primary breast cancer or other malignancies were excluded from the study. Accordingly, 305 patients represented the final cohort for the study, 154 of whom (50.4%) had lymph node involvement (LN+) at diagnosis. FFPE tissue sections of the primary tumor obtained after surgery were used. Clinical information was obtained from medical records. Tumors were reviewed and histologically classified according to the World Health Organization (WHO 2003) [27], graded using Elston and Ellis grading system [28] and grouped into stages according to TNM classification [29]. The patients’ cohort was followed for a maximum of 25 years through the local Cancer Registry from diagnosis of BC to death or until censoring date. This study was approved by the Ethical Committee of the University of Trieste, as already reported [4].

Patients were treated with mastectomy or breast-conserving surgery. All patients submitted to conserving surgery were treated with radiotherapy. All LN+ patients were treated with adjuvant chemotherapy, as already reported [4]. ER-positive patients, both LN− and LN+, were submitted to tamoxifen therapy. No specific treatment with trastuzumab was performed in HER2-positive patients, because this therapy was not available at the time of diagnosis.

### Tissue microarray

Tissue Micro Array (TMA) were built as already described (Pracella *et al.*, 2013) [4]. Briefly, tissues’ cores were chosen at the border of the primary tumor. TMA with tissue cores of 1.0 mm in diameter were built using a tissue-arrayer (Galileo TMA CK3500; Integrated Systems Engineering, Milano, Italy), as previously described [30]. Six TMA blocks containing up to 60 tissue cores each, were obtained. Multiple samples were taken for cases as representative of heterogeneous histological areas and considered positive if at least one core was positive for the specific biomarker. For each TMA block, 4 μm thick sections were cut, mounted on Superfrost^®^ Plus (Thermo Scientific) microscope slides and heated at 37°C overnight for IHC analysis.

### Immunohistochemical staining

IHC staining was performed following standard procedures [31], according to the manufacturers’ instructions for α-L-1–Fucosidase (FUCA-1) Polyclonal Antibody (Proteintech, Chicago, USA). Immunostaining was performed manually with the Vectastain Universal Elite ABC kit (Vector Laboratories, Burlingame, CA, USA). Polyclonal antibody diluted 1:50 was applied for 45 minutes after 20 minute antigen retrieval at pH8 using W-CAP TEC buffer (Bioptica, Milano, Italy) at high temperature, in water bath. For visualization, the DAB Substrate kit for Peroxidase (Vector Laboratories, Burlingame, CA, USA) was used. Molecular subtyping of the entire cohort has been previously performed by IHC using ER, PR, HER2 and Ki67 antibodies as described elsewhere [4].

### Evaluation of immunohistochemistry

Immunostaining was evaluated by two different observers in a blinded fashion (GS, RB), using light microscopy. Cytoplasmic staining was recorded and analyzed. Any positive cytoplasmic staining in tumor cells was considered as positive expression, with a cut-off of 10% cells. Tumors were classified into four main molecular classes according to the staining profile of the antigen markers as already reported [1, 4], notably luminal A and B, Her2 overexpressing, and (TN). If the tumors exhibited markers staining that did not meet the above-mentioned panel criteria, they were defined as ‘unclassified’.

### RT-PCR assay

FUCA-1 gene expression was quantitatively measured by Real Time-PCR (qRT-PCR). Briefly, total RNA was extracted from FFPE tissues after TMA microdissection, as previously described [32]. For each sample 4 μg of total RNA were treated with DNase, as described [33]. Complementary DNA synthesis was performed from 1.2 μg RNA, using Moloney Murine Leukemia Virus (M-MLV) reverse transcriptase (Invitrogen, Karlsruhe, Germany) by random hexamers priming in a final volume of 20 μl, as described elsewhere [34]. Expression levels of FUCA-1 and β-Actin genes were analyzed by real-time PCR using a Mastercycler^®^ ep Realplex (Eppendorf, Hamburg, Germany). PCR assays were performed in duplicates using the JumpStart™ Taq ReadyMix™ for Quantitative PCR (Sigma-Aldrich, St. Louis, MO, USA) according to the manufacturer’s instructions. In each run, 30 ng of cDNA for β-Actin and 135 ng for FUCA-1 were amplified in a final volume of 20 μl. Cycle conditions are reported in Supplementary Table 5. To exclude contamination, negative controls without cDNA were included in every run. RNA extracted from the breast cancer cell line MCF-7 was used as positive control. Primer and probe sequences, created by the use of the Primer Express software (Applied Biosystem, Darmstadt, Germany) and Primer3 (http://primer3plus.com/primer3web/primer3web_input.htm), are reported in Supplementary Table 5. Gene expression levels were normalized using β-Actin as reference gene, while a mixture of six samples of the cohort were pooled and used as calibrator. The relative quantitation was determined using the method proposed by Pfaffl [35]. For FUCA-1 determination in cultured cell lines, total RNA was isolated with the RNeasy Kit (Qiagen, Crawley, West Sussex, UK). One μg of RNA from each sample was reverse-transcribed with the QuantiTect^®^ Reverse Transcription kit (Qiagen). PCR reactions were performed in triplicates and fold changes were calculated with the formula: 2- (sample 1 ΔCt - sample 2 ΔCt), where ΔCt is the difference between the amplification fluorescent thresholds of the mRNA of interest and the mRNA of β-Actin used as an internal reference.

### Cultured cell lines

Breast adenocarcinoma luminal A cells (MCF-7 and T47D), luminal B (BT474), HER2+ (SK-BR3) and TN (MDA-MB231) cells were obtained from Dr. Aniello Cerrato (IEOS/CNR) and Prof. Roberto Bianco (University of Naples Federico II). TPC1 cells, derived from a papillary thyroid carcinoma, were obtained by M. Nagao (Carcinogenesis Division, National Cancer Center Research Institute, Tokyo, Japan). Cells were grown in Dubecco’s modified MEM (DMEM) or RPMI1640 medium supplemented with 10% fetal bovine serum (FBS). Media were supplemented with 2 mM L-glutamine and 100 units/ml penicillin-streptomycin (GIBCO).

### Antibodies

Anti-FUCA-1 is a polyclonal antibody from Proteintech (16420-1-AP) which recognizes the α-L-1-Fucosidase protein. Monoclonal anti-β-tubulin (#T9026) antibody was from Sigma Aldrich (St Louis, MO, USA). Secondary antibodies coupled to horseradish peroxidase were from Amersham Pharmacia Biotech (Piscataway, NJ, USA).

### Immunoblotting

Protein lysates were prepared according to standard procedures. Briefly, cells were harvested in lysis buffer (50 mM Hepes, pH 7.5, 150 mM NaCl, 10% glycerol, 1% Triton X-100, 1 mM EGTA, 1.5 mM MgCl2, 10 mM NaF, 10 mM sodium pyrophosphate, 1 mM Na3VO4, 10 μg of aprotinin/ml, 10 μg of leupeptin/ml) and clarified by centrifugation at 10,000 × *g*. Protein concentration was estimated with a modified Bradford assay (Bio-Rad) and lysates were submitted to Western blot. Membranes were probed with the above mentioned antibodies. Immune complexes were revealed by an enhanced chemiluminescence detection kit (ECL, Amersham Pharmacia Biotech). Signal intensity was quantitated using a Phosphorimager (Typhoon 8600, Amersham Pharmacia Biotech) interfaced with the Image Quant software.

### Statistical analysis

Associations between clinical-pathological factors and categories of markers were tested for significance using the chi-square test (or Fisher’s exact test, depending on sample size) for categorical variables. Cancer specific survival (CSS), defined as the lapse of time between the date of diagnosis and the date of BC specific death or the end of follow-up (FU) was the end point evaluated in this study. The log-rank test and Kaplan-Meyer curves were used to check the dependence of patients’ survival on single variables. To estimate the joint effects of the analysed covariates on patients’ survival and confirm the results of the log-rank test, data were analyzed by fitting Cox proportional hazard regression model. Cox analysis included pathological variables (histologic type of tumor, tumor grade, tumor size, number of positive lymph nodes and age at diagnosis) and molecular markers which resulted previously significant at log-rank test. All *p*-values are two-sided with values < 0.05 regarded as statistically significant. *P*-values between 0.05 and 0.07 were considered as “borderline”. Statistical analyses were performed with the Stata/SE 12 package (Stata, College Station, TX).

## SUPPLEMENTARY MATERIALS FIGURES AND TABLES


