# Supplementary material for: Reduced expression of α-L-Fucosidase-1 (FUCA-1) predicts recurrence and shorter cancer specific survival in luminal B LN+ breast cancer patients
Source: Oncotarget. 2018 Feb 7;9(20):15228–38. doi: 10.18632/oncotarget.24445 (PMC5880599; doi:10.18632/oncotarget.24445)
Supplement: Supplementary file 1 [file oncotarget-09-15228-s001.pdf]

Reduced expression of α-L-Fucosidase-1 (FUCA-1) predicts recurrence and shorter cancer specific survival in luminal B LN+ breast cancer patients

SUPPLEMENTARY MATERIALS

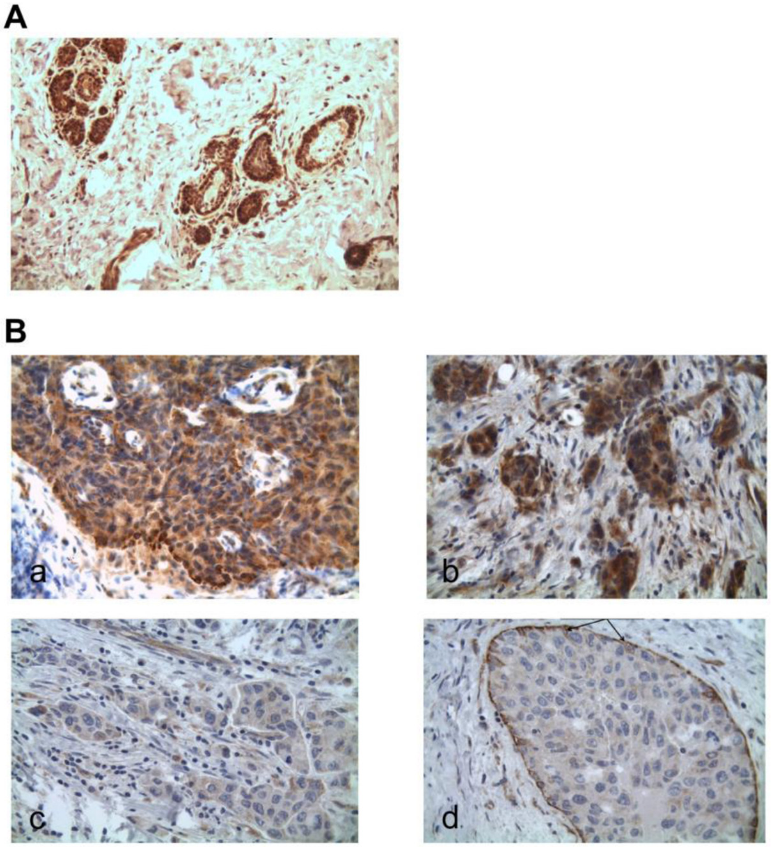

Supplementary Figure 1: Immunohistochemical staining for FUCA-1 of an archival section from a normal atrophic mammary gland (**A**), from a well differentiated breast cancer (**B** a), from a breast cancer having an intermediate degree of differentiation (**B** b), from a poorly differentiated breast cancer (**B** c) and from an intraductal breast carcinoma (**B** d). Magnification 10×. The arrows point to the staining of myoepithelial cells.

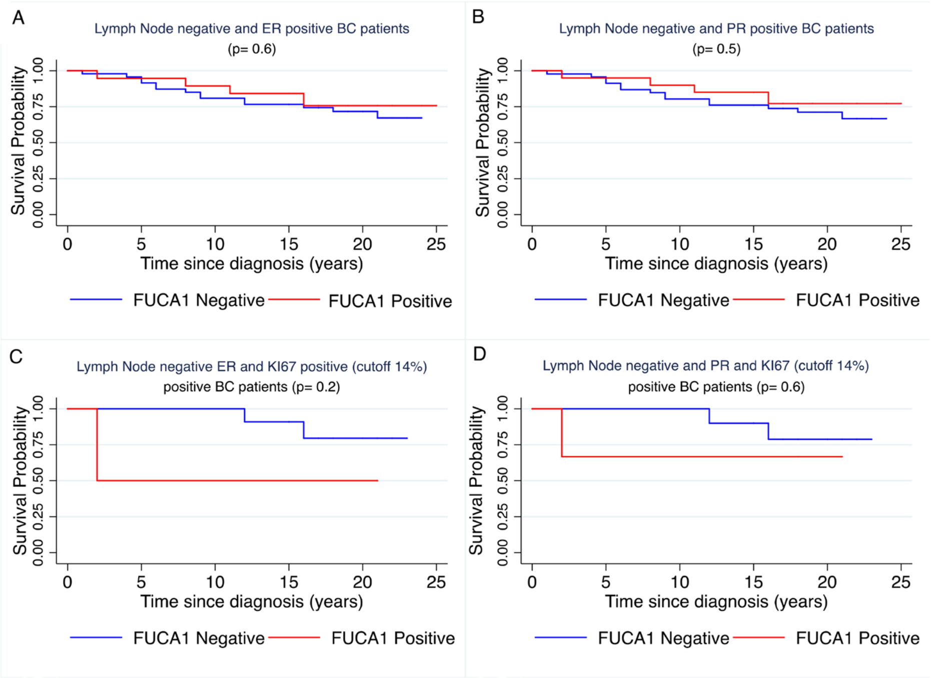

Supplementary Figure 2: Kaplan-Meier curves of cancer specific survival by positivity or negativity to FUCA-1 immunostaining in LN– ER+ patients (**A**), in LN–, PR+ patients (**B**), in LN–, ER+ patients with Ki67 staining higher than 14% (**C**) and in LN–, PR+ patients with Ki67 staining higher than 14% (**D**).

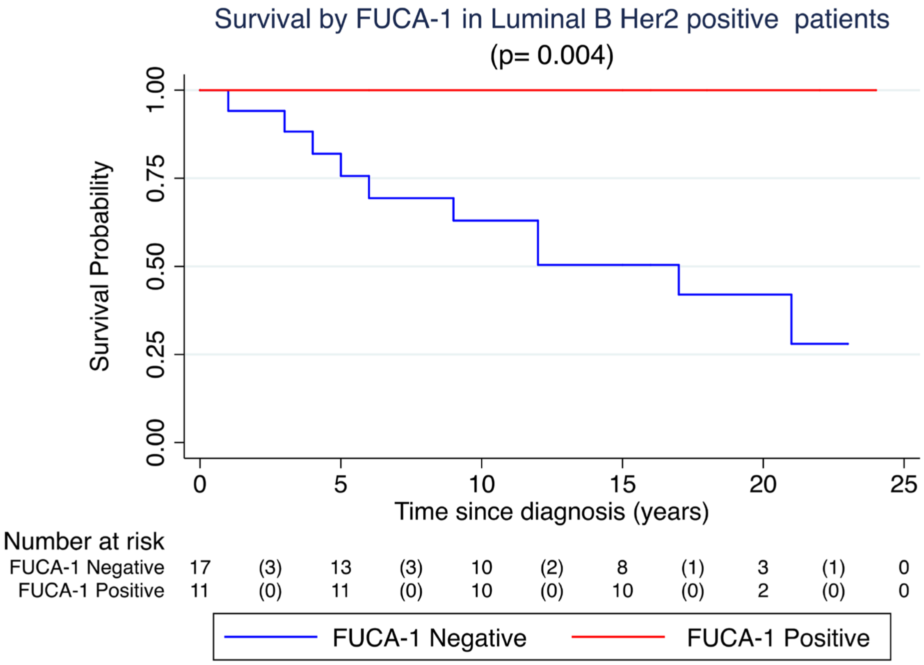

Supplementary Figure 3: Kaplan-Meier curves of cancer specific survival by positivity or negativity to FUCA-1 immunohistochemical staining in Luminal B HER2+ patients. In life table are reported at risk patients and in brackets the breast cancer specific deaths for the specific time interval.

Supplementary Table 1: Clinical and pathological characteristics of the breast cancer patients who were successfully investigate for FUCA-1

Factors	Total case-study 204 (100%)	LN– 83 (41%)	LN+ 121 (59%)
**Age, years**			
≤35	14 (6.9%)	1 (2.6%)	13 (10.7%)
>35	190 (93.1%)	82 (97.4%)	108 (89.3%)
**Mean Age , years (range)**	47.2 (26-55)	46.9 (32-55)	47.4 (26-55)
**Histology**			
Ductal	167 (81.9%)	61 (73.5%)	106 (87.6%)
Lobular	18 (8.8%)	7 (8.4%)	11 (9.1%)
Medullary	5 (2.5%)	5 (6.0%)	-
Mucinous	7 (3.4%)	4 (4.8%)	3 (2.5%)
Tubular	7 (3.4%)	6 (7.2%)	1 (0.8%)
**Grade**			
1	24 (11.7%)	19 (22.9%)	5 (4.1%)
2	92 (45.1%)	44 (53%)	48 (39.7%)
3	88 (43.1%)	20 (24.1%)	68 (56.2%)
**Tumor size, cm**			
≤2	116 (57.1%)	60 (72.3%)	56 (46.7%)
2–5	77 (37.9%)	22 (2.5%)	55 (45.8%)
≥5	10 (4.9%)	1 (1.2%)	9 (7.5%)
**Lymph nodes**			
1–3 lymph nodes	77 (37.7%)	-	77 (63.6%)
≥4 lymph nodes	43 (21%)	-	43 (35.5%)
Unknown	1 (0.8%)		1 (0.8%)
**Stage**			
I	59 (29%)	59 (71.1%)	-
II	90 (44%)	23 (27.7%)	67 (55.4%)
III	54 (26.5%)	1 (1.2%)	53 (43.8%)
Unknown	1 (0.5%)	-	1 (0.8%)
**Recurrence**			
No	88 (43.1%)	48 (57.8%)	40 (33.1%)
Yes	105 (51.5%)	32 (38.6%)	73 (60.3%)
Unknown	11 (5.4%)	3 (3.6%)	8 (6.6%)
**ER**			
Negative	46 (22.6%)	17 (20.5%)	29 (24. %)
Positive	158 (77.4%)	66 (79.5%)	92 (76%)
**PR**			
Negative	59 (28.9%)	17 (20.5%)	42 (34.7%)
Positive	145 (71.1%)	66 (79.5%)	79 (65.3%)
**Her-2**			
Negative	156 (76.2%)	74 (89.2%)	82 (67.8%)
Positive	48 (23.5%)	9 (10.8%)	39 (32.2%)
**Ki67**			
<14%	95 (46.6%)	54 (65.1%)	41 (33.9%)
≥14%	109 (53.4%)	29 (34.9%)	80 (66.1%)
**Molecular Subtypes**			
Luminal A	83 (40.7%)	51 (61.5%)	32 (26.5%)
Luminal B	75 (36.8%)	15 (18.1%)	60 (49.6%)
Her2 non Luminal	20 (9.8%)	5 (6%)	15 (12.4%)
TN	24 (11.8%)	11 (13.2%)	13 (10.7%)
Unknown	2 (1%)	1 (1.2%)	1 (0.8%)

Supplementary Table 2: Protective effect of FUCA-1 expression in LN+ luminal B patients using the covariates of stage, grade, age at diagnosis and histological type of tumor

Variable_	Haz. Ratio	Standard Err.	*P* > z	95% Conf. Interval
Stage	.9356486	.3536163	0.860	.446–1.96
Grade	1.142308	.3761113	0.686	.599–2.18
Age at diagnosis	.992387	.0274734	0.783	.940–1.05
Histologic type	.9495623	.3470957	0.887	.464–1.94
FUCA-1 cytoplasmatic	.2513416	.1302586	0.008	0.091–0.694

General result of regression: $p = 0.04$.

Supplementary Table 3: Effect of ER, PR, Ki67 on survival with respect to cytoplasmic expression of FUCA-1 in LN+ BC patients

Variables				*p* value
**Ki67 (cut-off 14%)**	**ER**	**PR**	**HER2**	
x				0.14
	x			0.03
		x		0.02
x	x			0.001
x		x		0.0009
			x	0.04
x			x	0.09
	x		x	0.005
		x	x	0.009

p values refer to log-rank test obtained for cytoplasmic expression of FUCA-1 in LN+ BC patients positive for the marked biomarkers. For example, the *p* value of 0.14 refers to log-rank test calculated for cytoplasmic expression of FUCA-1 in LN+, Ki67+ (cut-off 14%) BC patients, *p* value of 0.03 refers to log-rank test calculated for cytoplasmic expression of FUCA-1 in LN+, ER+ BC patients, $p = 0.02$ is related to log-rank test calculated for cytoplasmic expression of FUCA-1 in LN+, PR+ BCt cancer patients; $p = 0.01$ refers to log-rank test calculated for cytoplasmic expression of FUCA-1 in LN+, Ki67+ (cut-off 14%) and ER+ BC patients.

Supplementary Table 4: Data from oncomine [1]

Cancer type	Cell line	FUCA-1 Log2 median-centered intensity
Breast carcinoma	DU-4475	7,871
Breast carcinoma	EVSA-T	4,754
Breast carcinoma	MDA-MB-453	4,148
Metablastic breast carcinoma	HCC1569	4,806
Breast adenocarcinoma	CAL-85-1	7,055
Breast adenocarcinoma	CAL-51	6,656
Breast adenocarcinoma	AU565	6,388
Breast adenocarcinoma	EFM-192A	5,845
Breast adenocarcinoma	MDA-MB-361	4,528
Breast adenocarcinoma	CAL-120	4,281
Ductal breast carcinoma	KPL-1	5,722
Ductal breast carcinoma	ZR-75-1	5,591
Ductal breast carcinoma	JIMT-1	3,187
Ductal breast carcinoma	HCC1428	1,361
Invasive ductal breast carcinoma	HCC1419	7,472
Invasive ductal breast carcinoma	CAL-148	5,949
Invasive ductal breast carcinoma	HCC2218	5,340
Invasive ductal breast carcinoma	HCC1937	4,997
Invasive ductal breast carcinoma	HCC1954	4,885
Invasive ductal breast carcinoma	BT-549	4,589
Invasive ductal breast carcinoma	HCC11433	3,734

Supplementary Table 5: Sequence of primers and cycling conditions used for q-RT-PCR experiments

Gene	Primers sequences	Cycling conditions
β-Actin	Fw primer:<br>5′-GTGGATCAGCAAGCAGGAGT-3′<br>Rev primer:<br>5′-AGGGTGTAACGCAACTAAGTCA-3′<br>Probe (M<br>5′-/FAM/CAC CGC AAA TGC TTC-3′	PCR: 2 steps:<br>Denaturation 95° C, 10 min<br>40×: 95° C/15 sec; 60° C/1 min
FUCA-1	Fw primer:<br>5′-ATG GAC TGA TTG TTC CCA TCT T-3′<br>Rev primer:<br>5′-CCA TGG TTT GGA GGC ATA GA-3′<br>Probe:<br>5′-FAM/AGC CAT TTC /ZEN/CCA ACA GCA AGA AGC-3′	PCR: 3 steps:<br>Denaturation 95° C, 2 min<br>45×: 95° C/15 sec; 56° C/30 sec;<br>72° C/30 sec

REFERENCE

1. Adai A. Breast cancer cell lines. Genentech, not published, https://www.ncbi..nlm.nih.gov/.
